# Bioreactor-Scale Strategies for the Production of Recombinant Protein in the Yeast *Yarrowia lipolytica*

**DOI:** 10.3390/microorganisms7020040

**Published:** 2019-01-30

**Authors:** Marie Vandermies, Patrick Fickers

**Affiliations:** TERRA Teaching and Research Centre, Microbial Processes and Interactions, University of Liège–Gembloux AgroBio Tech, 5030 Gembloux, Belgium; marie.vandermies@doct.uliege.be

**Keywords:** *Yarrowia lipolytica*, recombinant protein, bioreactor, metabolic load

## Abstract

Recombinant protein production represents a multibillion-dollar market. Therefore, it constitutes an important research field both in academia and industry. The use of yeast as a cell factory presents several advantages such as ease of genetic manipulation, growth at high cell density, and the possibility of post-translational modifications. *Yarrowia lipolytica* is considered as one of the most attractive hosts due to its ability to metabolize raw substrate, to express genes at a high level, and to secrete protein in large amounts. In recent years, several reviews have been dedicated to genetic tools developed for this purpose. Though the construction of efficient cell factories for recombinant protein synthesis is important, the development of an efficient process for recombinant protein production in a bioreactor constitutes an equally vital aspect. Indeed, a sports car cannot drive fast on a gravel road. The aim of this review is to provide a comprehensive snapshot of process tools to consider for recombinant protein production in bioreactor using *Y. lipolytica* as a cell factory, in order to facilitate the decision-making for future strain and process engineering.

## 1. Introduction

Recombinant protein production represents a multibillion-dollar market [1,2]. It has been developed as a safer and cost-effective alternative to the extraction of proteins from natural sources. Recombinant protein production is mainly devoted to biopharmaceuticals and industrial enzymes (e.g., for the food, feed, detergent, paper, biofuels, and fine chemical industries). As unicellular eukaryotes, yeasts offer a convenient middle ground recombinant expression system between limited prokaryote hosts and delicate higher eukaryote hosts. Yeasts indeed combine the characteristics of the former (i.e., simplicity of growth and genetic engineering) and the latter (i.e., protein folding and assembly, post-translational modifications).

In this regard, *Yarrowia lipolytica* stands out as an advantageous host system. This yeast has been recognized by the American Food and Drug Administration (FDA) as an organism generally regarded as safe (GRAS status), making it suitable for food and pharmaceutical applications [3]. Compared to other host organisms such as *S. cerevisiae*, *Y. lipolytica* encounters reduced hyperglycosylation issues, narrowing its glycosylation patterns from mammalian ones [2]. In its natural environment, *Y. lipolytica* secretes large amounts of hydrolases, mainly lipases and proteases, to retrieve nutrients necessary to sustain its growth. This goes together with a very efficient secretory pathway [4]. *Y. lipolytica* strains have been isolated from diverse environments such as sea water, cheese, or waste sludge [5], explaining this yeast adaptability to a wide range of substrates and culture conditions.

**Figure 1:**
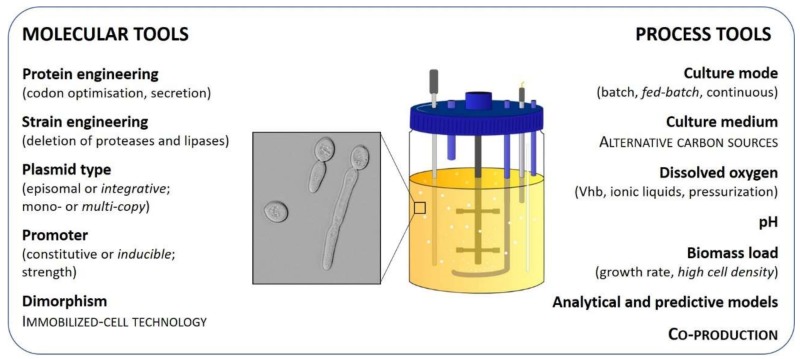
Molecular and process tools available for bioprocess optimization, as developed in the text below. Bold font: main strategies; normal font: available options; italic font: preferred options; capital font: promising cost-effective options.

To date, more than 150 recombinant proteins have been produced using *Y. lipolytica* as a cell factory. However, only 25 of them have been produced at a bioreactor scale. Compared to *Pichia pastoris*, occurrences of *Y. lipolytica-*based processes are still scarce at the industrial scale, despite similar yield and productivity ranges. Regardless of reactor-scale recombinant production advances in *Y. lipolytica*, process efficiency is still hampered by issues such as metabolic load, unmastered dimorphism, and oxygen requirements. Molecular and process strategies (summarized in Figure 1) offer solutions to consequently adjust recipient strains and cultivation conditions, in order to optimize protein quality as well as volumetric yields and productivity [1], the key parameters for bioprocess economic feasibility. The aim of the present review is to provide a comprehensive snapshot of the already available and future molecular and process tools to consider for recombinant protein production in bioreactor using *Y. lipolytica* as a cell factory, in order to facilitate decision-making for future strain and process engineering.

## 2. Protein Production

### 2.1. Recombinant Proteins

Aside from overexpressed endogenous genes, reactor-scale production by *Y. lipolytica* has mainly focused on recombinant proteins from fungal origin, although some examples of viral [6], bacteria [7,8,9,10,11], vegetal [12,13,14,15], or mammalian [16,17,18,19,20] proteins have been reported over the years. These recombinant proteins are mainly enzymes (secreted or intracellular) that modify the properties of macromolecules (hydrocarbons, proteins, and lipases) for applications in food and feed, cosmetics, detergents, textiles and paper industries, biofuels, and waste stream depollution. A second, narrower category concerns the medical and pharmaceutical fields. Heterologous enzymes produced in *Y. lipolytica* can serve to obtain enantiopure molecules suited for drug synthesis [21,22,23]. Hyperglycosylation, commonly observed in *S. cerevisiae*, is not critical in *Y. lipolytica* [24,25], rendering this host more adapted for the production of therapeutic proteins such as virus antigen [6], glucocerebrosidase [26], and lysosomal enzyme [20]. Strains with modified glycosylation pathway, the so-called humanized strains, allow glycosylation patterns closer to that of human glycoproteins [27]. Finally, some heterologous proteins are produced for the sake of process improvement. This is notably the case for the single-chain hemoglobin Vhb from *Vitreoscilla stercoraria* [7], which promotes cell growth in an oxygen-limited environment, the invertase Suc2p from *S. cerevisiae* [28,29], or the exo-inulinase Inu1p from *Kluyveromyces marxianus* [30], converting respectively sucrose and inulin into glucose and fructose that can further be metabolized by the cells. Reporter genes, such as those encoding β-galactosidase [8,10] or yellow fluorescent protein (YFP, [31]), allow one to monitor the regulation of inducible promoters during bioreactor operations.

### 2.2. Metabolic Load: the Weight of Recombinant Expression on Host Cells

Following the overexpression of a recombinant gene in a host cell, resources such as energy, transcription and translation factors, and amino acids will be allocated to maintenance of the replicative plasmid used to host recombinant gene (if applicable) and to recombinant protein synthesis and secretion at the expense of cell metabolism, causing physiological disorder. The metabolic load corresponds to the proportion of resources diverted from endogenous cellular mechanisms to recombinant gene maintenance and expression [32,33]. It has been found that metabolic load increases with increasing recombinant gene size [33,34] and copy number (see below), and is further amplified by the expression level [32]. Environmental factors such as limited amounts of nutrients and low dissolved oxygen levels enhance the difficulty for cells to cope with metabolic load [32,35].

The most evident effect of this metabolic load is the reduced growth capacity of host cells. Specific growth rate and biomass yield both suffer from resource diversion [32,33,36,37]. On a molecular level, limitations in specific amino acids and energy provision may result in translational errors, impacting recombinant protein activity, stability, and possible immunogenicity [32]. Moreover, increased heterogeneity in protein population quality highly complexifies their purification, and must consequently be avoided at an industrial scale, especially for pharmaceutical applications. Additionally, recombinant proteins directed to the secretory pathway compete with essential endogenous proteins for exportation (i.e., RNases, lipases, esterases, proteases, and phosphatases [38]), and may trigger the unfolded protein response, initiating additional stress events in protein folding, vesicular transport, and protein degradation [39,40] that will further contribute to metabolic load [41]. Cell fitness may further be impaired by the activity of certain heterologous proteins, interfering with host cell functioning [32,42].

To alleviate the metabolic load afflicting recombinant host cells, molecular and process strategies must be implemented. Integration of expression cassette in yeast chromosome shall be favored over episomal plasmids, whose maintenance in the cells requires a substantial amount of energy. When codon bias differs significantly between heterologous gene and recipient cells, the synthesis of a codon-optimized gene variant can be considered [16,17,20,37,43]. Codon usage frequency of the synthetic gene shall reflect that of the native producer to allow fluent elongation on the ribosome and correct protein folding [44]. Improvement of secretion efficiency [45] and synthesis of pro-proteins [11,19] will further help the cells to tolerate overall expression pressure. Determination of an optimal growth rate—not always corresponding to the maximum growth rate µ_max_—during recombinant protein production promotes balance between growth and protein synthesis, especially for constitutive promoters [23]. At the process scale, inducible promoters exploited in a fed-batch mode allow biomass to grow at a high growth rate (close to µ_max_) prior to the initiation of the production phase. Moreover, regulation of the expression level via dose-dependent induction is a promising exploratory path to modulate the metabolic load. Medium optimization, fed-batch, and improved dissolved oxygen management help ensure sufficient nutrient and oxygen supply to recombinant cells enduring metabolic load.

## 3. Molecular Strategies

### 3.1. Strains and Plasmids: Backbones of Recombinant Expression

The history of recombinant protein production in *Y. lipolytica* started with “homemade” host strains, varying from one laboratory to the other, hence hardly comparable. The construction of the Po1 series of optimized strains for heterologous protein production constitutes a milestone, since these strains are among others deleted for extracellular protease genes *AEP* and/or *AXP*, and can grow on sucrose as a sole carbon source (for details, see [19,46]). For recombinant lipase production and exploitation of lipid-inducible promoters, additional engineering delivered strains devoid of intrinsic lipase activity. The strain MTLY50 is deleted for *AEP* gene and *Y. lipolytica* main lipase *LIP2* gene [47], whereas the strain JMY1212 is additionally deleted for *LIP7* and *LIP8* genes [47,48]. For recombinant therapeutic protein production, decreased glycosylation and/or glycosylation patterns closer to mammalian ones are enabled by the disruption [26,49,50,51,52] or overexpression [49] of genes related to glycosylation. These genetic modifications have been gathered in the glyco-engineered strains patented by Universiteit Gent (Gent, Belgium), Vlaams Instituut voor Biotechnologie (Gent, Belgium), and Oxyrane UK Ltd. (Manchester, UK) in 2011 [27].

In the same line, a panel of optimized plasmids for expression of heterologous genes were developed (for details, see [19,53]), from which one plasmid has been commercialized in an expression kit (Yeastern Biotech, Taipei, Taiwan). All of them bear integration sequences that allow stable integration in the yeast genome. Indeed, episomal plasmids do not yield higher protein production level in the bioreactor [14] since they require the maintenance of a selection pressure quickly becoming cumbersome in large-scale and/or prolonged cultivations, and are less stable in any case [14,43]. The established integration methods comprise random zeta integration (the resulting expression may vary among transformants according to the integration locus of the expression cassette) or site-directed integration using the pBR322 or zeta docking platform. Newly developed tools will greatly facilitate integration, namely the CRISPR-Cas9 system for markerless integration [54,55], the *∆ku70* strain for improved homologous end joining [56], and targeted integration in intergenic sites with high gene expression levels [57]. Upcoming studies shall help with evaluations of engineered strain robustness and adequacy with respect to reactor cultivations.

### 3.2. Copy Number: Is More Better?

Regarding the copy number of recombinant genes integrated in the yeast genome, mono-copy expression stays predominant in the reported reactor-scale productions. Multi-copy may be hampered by genetic stability issues, globally increasing with the number of copies integrated in the genome, and is further influenced by the culture conditions, recipient strain, integration locus, and individual cell behavior [46,58]. Indeed, cells intend to get rid of exogenous genetic elements non-essential to their survival in given circumstances. However, multi-copy proved to be a rewarding strategy to improve process performance, as detailed below. When comparing multi-copy versus mono-copy variants in a batch bioreactor, the generally observed trend is a net increase of recombinant protein yields and productivity, at the expense of cell growth, which reflects the phenomenon of metabolic load. Nicaud and colleagues performed the first reported reactor application of the defective marker *ura3d4* [46] for leucine amino peptidase production. The use of a multi-copy strain yielded a 7-fold increased specific enzyme production rate and 8-fold increased final enzyme activity. The cells reached the same final concentration in both cases, with the multi-copy variant growing noticeably slower [53]. This pattern was later confirmed for fed-batch mannanase production. Comparing a mono-copy strain fitted with a *LIP2* secretion signal to 8- and 9-copy strains with a native secretion signal, Roth and colleagues noticed a biomass decrease exacerbated by the copy number. Still, the volumetric and specific glucose utilization rates increased with the copy number, revealing that glucose was directed toward endo-1,4-β-mannanase synthesis at the expense of biomass production. Indeed, for strains bearing the 8- and 9-copy of the expression cassette, the volumetric enzyme yields reached respectively 8- and 10-fold the mono-copy strain one, and the same trend was observed for enzyme productivity [36]. YaPing and colleagues constructed mono- and multi-copy (6 copies) strains to produce in a batch bioreactor two mannanases, namely manA from *Aspergillus niger* and manB from *Bacillus subtilis*. Compared to mono-copy integration, multi-copy strategy resulted in a 3.5-fold and 4.5-fold increased enzyme yield and productivity for manA and manB, respectively. By contrast, biomass yield was reduced by 2.2- and 1.9-fold, respectively [37]. The only report on a positive effect of multi-copy integration on biomass growth is for *SUC2* gene encoding invertase in sucrose-based batch, whose double integration resulted in a higher growth rate (0.132 versus 0.096 h^−1^) along with an improved enzyme yield (1991 versus 597 U·L^−1^) and productivity (27.65 versus 8.29 U·L^−1^·h^−1^). This can be explained by the enhanced ability to metabolize the available carbon source conferred by a higher invertase activity [29]. Further development of multi-copy strategies at a large scale will require overcoming copy number instability issues and will indicate the clearest proportional relationship possible between copy number and expression intensification (which also depends on a host of other factors relative to the recombinant protein, recipient strain, and plasmid as well as to the process parameters).

### 3.3. Promoters: an Inducible Advantage

Expression pattern and efficiency rely on the selected promoter. The promoter of the *AEP* gene encoding alkaline extracellular protease, namely *pXPR2*, was developed and exploited in the first attempts to control recombinant expression in *Y. lipolytica* [6,59,60,61] and has been patented by Pfizer Inc. in 1993 [62]. *pXPR2* is a strong promoter, induced by high peptone concentrations at a pH above 6 [63]. These specific conditions for induction led to the design of pioneer fed-batch strategies, detailed hereafter. They also fostered the development of more polyvalent promoters whose applications were not limited to rich, peptone-containing media [58]. One of them, named *hp4d*, is a synthetic promoter combining four copies of the upstream activating sequences (UASs) of *pXPR2* (UAS1_XPR2_) located upstream of a minimal *LEU2* promoter (core element) [19]. Though induction levels obtained with this promoter were encouraging, *php4d*-driven expression was found to be growth-phase dependent, which is less compatible with some industrial applications [53]. Another promoter, derived from translation elongation factor-1α, *pTEF*, was found uncoupled from cell growth phase and yielded induction level comparable to *php4d* [64]. The construction of synthetic promoters were pursued with up to 32 copies of a sequence-optimized derivative of UAS1_XPR2_ upstream of a *LEU2* or *TEF* core element, and promoter strength was found correlated with the number of UASs [65]. This synthetic promoter design was then extended to UASs and core elements of other promoters [65,66,67,68]. Constitutive high-level expression frequently exhausts the host cells [32], explaining why inducible systems appear preferable for recombinant protein production [2]. In that respect, alternative inducible promoters to *pXPR2* were proposed to dissociate the growth and production phases, and thereby alleviate the metabolic load imposed by recombinant expression.

Among the developed promoters responding to hydrophobic inducers [53,69], applications based on *pPOX2* (promoter of acyl-CoA oxidase) and *pLIP2* (promoter of lipase Lip2p) reached the reactor scale. The former served essentially to express fungal lipase genes [70,71] and human proteins following oleic acid induction [16,17,18,20], in both complex and defined media, while the latter has so far been limited to a reporter system [8,10] and lipase Lip2p recombinant production [72]. *pLIP2*-driven expression appears more sensitive than *pPOX2* to environmental conditions, in the sense that it is influenced by pH and nitrogen source nature and concentration. With *pLIP2*, higher protein yields are obtained at pH 6 over pH 3 in a defined medium [73], or with a sufficient load of tryptone N1 in a complex medium [72,74]. In both systems, complete discrimination of growth and induction phases is prevented by *Y. lipolytica* consumption of some hydrophobic inducers (e.g., triglycerides) as a carbon source. Host strains deleted for *Y. lipolytica* main lipase genes, namely *LIP2*, *LIP7*, and *LIP8* [47,48,75], overcome this issue. Moreover, disruption of lipase-encoding genes prevents host cell diversion from recombinant expression and avoids the presence of undesired proteins (i.e., lipases) in the culture supernatant [16,71]. Notwithstanding suboptimal strain background, both *pPOX2* and *pLIP2* delivered high expression levels in reported reactor studies. *pLIP2* would nevertheless be more efficient than *pPOX2* according to flask experiments [72,76]. The main drawback of hydrophobic inducers is their immiscibility with culture medium of aqueous nature, requiring the generation of an emulsion that becomes problematic to maintain during scale-up. In this regard, co-substrate feeding with a hydrophilic carbon source (e.g., glucose and glycerol) helps to reduce the dependency toward hydrophobic substances [76], but this strategy has not been implemented in reactor cultures yet.

Promoters responding to hydrophilic inducers offer a practical alternative to *pPOX2* and *pLIP2*. In this perspective, the characterization and functional dissection of upstream regions from the gene *EYK1* and *EYD1*, involved in erythritol and erythrulose metabolism in *Y. lipolytica*, led to the release of a series of engineered promoters with tunable strength (depending on the number of UASs), inducible by erythritol and erythrulose and repressed by glycerol and glucose [31,77]. With these patented promoters [78], erythritol and erythrulose can be used as an inducer and a carbon source in the wild-type strain, or solely as an inducer in the *Δeyk1* strain. During the characterization of a *pEYK1*-derived promoter in a bioreactor operated in chemostat mode, the promoter was found to respond to erythritol and erythrulose in a dose-dependent manner [31], paving the way to a fine regulation of metabolic load by gene titration. Yet, reactor observations were based on a reporter protein, i.e., a short, engineered YFP, optimized for expression in *Y. lipolytica*. Future developments shall opt for the production of a functional protein.

### 3.4. Unpredictable Dimorphism

*Y. lipolytica* is a dimorphic yeast, able to grow under morphologies ranging from ovoid, yeast-like cells to pseudohyphae to septate hyphae. Effectors of the dimorphic transition include pH, temperature, mechanical stress, osmotic pressure, carbon and nitrogen sources, buffer composition, and nutrient or oxygen deprivation (for a review, see [79]), whose influence varies according to the considered strain [80]. Dimorphic transition reflects profound physiological changes susceptible to affecting process outcomes, such as differential hydrocarbon degradation potential [81,82] and protein production capacity [17,83,84]. According to the considered bioprocess, higher yields may be reached with a hyphal ([83], for lipase) or ovoid ([17,84], for interferon and laccase, respectively) morphotype. Currently, *Y. lipolytica* morphological state is scarcely assessed and not efficiently mastered in a bioreactor. Individual cell behavior divergence, naturally occurring within a cell population, results in phenotypic heterogeneity, i.e., in a broad distribution of cell sizes and shapes which will in turn impact rheological properties of the culture broth, influencing heat and mass transfer phenomena [85,86]. Monitoring and control of cell morphology are thus essential to process optimization. So far, morphological observations have largely relied on offline tedious methods, mainly microscopy [87,88,89,90]. With the emergence of flow cytometry, *Y. lipolytica* cell shape distribution can be rapidly evaluated [73,91,92] and even monitored online [93]. From there, automated actuation could be implemented to control population morphological heterogeneity [94]. A simpler, cost-effective solution to master *Y. lipolytica* morphology in a bioreactor would be to arrest the cells in a defined morphotype, as has recently been suggested for fatty acid production improvement [95]. Aside from environmental effectors, several molecular mechanisms have been found involved in the dimorphic transition [96,97,98,99,100,101,102,103,104,105]. A variety of strains growing constitutively in the yeast-like form is available, namely *Δhoy1* [105], *Δmhy1* [99], *Δcla4* [106], *Δbem1* [100], *Δylbmh1* [101], and *Δste11* [97]. Provided that gene disruption does not alter other cell functions, ovoid mutants perfectly match the requirements of planktonic cultures in bioreactors. On the other hand, filamentous morphology confers to the cells an increased retention ability to a support inside the bioreactor, opening the path for immobilized-cell technology [107]. This technology has already proved its strength for succinic acid production [108,109] and could be advantageously applied to secreted recombinant proteins [107]. With immobilized-cell bioreactors, downstream processing is considerably simplified since cells are retained in the reactor, greatly reducing the bioprocess overall cost. Currently identified filamentous mutants, *Δtpk1* [96] and *Δylhls1* [107], can contribute to controlled cell immobilization inside bioreactors.

## 4. Process Strategies

### 4.1. The Predominance of Fed-Batch

Following a first process development phase centered on the batch process, most studies tend to optimization via fed-batch cultures, which ally the possibility of decoupling growth and production phases with a better support of cellular activity through a controlled nutrient uptake. Indeed, neither starving nor overfed cells are desirable, nor is a high nutrient concentration that could alter physical properties of the culture medium and impair cell growth or protein production. Reactor-scale continuous processes have been reported as exploratory chemostats aiming to assess the optimal conditions for promoter induction [13,31]. Semi-continuous cultures have been proposed with the idea of increasing productivity through time saving, for example the two-stage cyclic fed-batch process designed by Chang and colleagues for rice α-amylase production under the control of *pXPR2* [12,14]. In this process, portions of the culture broth are regularly transferred from growth to production medium (containing *pXPR2* inducer), while remaining cells in the growth phase are further fed with a growth medium. At the industrial scale, however, the risks associated with a continuous process—i.e., contaminations, strain mutations, and product instability—are too important to be taken. The only example of a continuous *Y. lipolytica*-based process implemented in the industrial practice was exploited for single-cell oil production from petroleum in the 1970s [38].

The transition from shake-flask to bioreactor cultures offers improved oxygenation conditions and medium homogeneity [16]. For culture scale-up from flask to pilot-scale reactors, a 3- to 5-fold higher enzyme yield, and an even higher productivity, can be expected as compared to shake-flasks [22,37,53,110,111]. For complex non-enzymatic therapeutic proteins, the gain can be even greater, with a 416-fold increase of human interferon concentration for a culture scale-up from a 250 mL shake-flask to a 5 L batch reactor [17]. The transition from batch to fed-batch mode drives the process further. Biomass concentration values in the range of 50–100 g dry cell weight (DCW)·L^−1^ are commonly attained with fed-batch processes [13,15,21,22,23,36,37,53,112], while protein yields and productivity thrive from 2–3-fold [37,113] to 10-fold [22,53].

Most fed-batch processes start with carbon source feeding [9,11,15,18,22,23,36,53] or complete culture medium feeding [13,14,113,114] at the end of a batch phase where the main carbon source has been depleted. This allows maintenance of the cell growth and metabolic activity, including recombinant protein production. For processes requiring the uncoupling of cell growth and the protein production phase, such a feeding can been executed during the growth phase only [71]. However, pursuing nutrient feeding during the production phase delivers higher biomass and protein yields, since the cells are supported in their growth-to-production-phase transition [18]. Exponential feeding models were established to satisfy precisely the nutritional needs of exponentially growing cells in a bioreactor [12,13,14,18,23,113]. These models aim to control the growth rate, to prevent cell overfeeding at the beginning of the culture and nutrient starvation during the exponential growth phase, and to avoid a negative influence on recombinant gene expression [23]. Currently, options such as one-step feeding [9,15], constant feed rates [21,53], and feed pulses at fixed times [28,71,112] are still preferred due to their convenience of implementation, particularly at a large scale, even if they deliver suboptimal recombinant protein productivity. Modeling approaches to control process productivity, reviewed recently for *P. pastoris* [115] could be applied in the future for *Y. lipolytica* now that genome-scale metabolic models of this yeast are available [116,117,118,119].

In fed-batch mode, inducible promoters are employed to dissociate the growth and production phases, in order to alleviate the metabolic load that recombinant protein synthesis brings on the host cells [16]. In the first instance, biomass is allowed to form to a concentration that sustains subsequent protein production [13,17,18,114]. An inducer is then fed to the culture medium, cell growth consequently slows down to the profit of protein synthesis, and metabolic fluxes are diverted from the former to the latter. This strategy was systematically implemented for proteins of human origin in reported studies [16,17,18,20], to balance the significant detrimental impact on cell fitness provoked by the initiation of human gene expression [18].

To unveil the regulation modalities of *pXPR2*, Chang and colleagues [13] exploited the principle of chemostat, which places the culture at the steady state. They consequently confirmed the optimal pH for *pXPR2* induction (i.e., 6.8) and determined the best nitrogen sources for induction (i.e., proteose peptone, and, to a lesser extent, neopeptone) and those acting as a repressor (ammonium sulfate and, to a lesser extent, casamino acids). They also defined the optimal ratio of carbon source to inducer to be used. These findings were then consistently applied in following studies [12,14]. Given that *Y. lipolytica* tolerates high osmotic pressure, a one-step addition of proteose peptone together with a concentrated glycerol solution was used as a simpler feeding strategy [15]. For an identical final biomass, enzyme yields were increased by 30% using an adequate proteose peptone concentration (2.0% instead of 0.25%). Except for a late study [28] confirming that a pH of nearby 6.8 is crucial for proper induction, the promoter *pXPR2* has slowly fallen into disuse, mainly because most of the bioreactor processes are based on a defined medium. 

Currently, induction of recombinant gene expression is operated under the control of *pPOX2* and *pLIP2*, even at an industrial scale [53]. To induce *pPOX2*, several oleic acid feeding strategies have been attempted, from a one-step addition [16,17] to periodic inducer pulses [18] and to constant [18] or gradually increasing [71] feed rates. The latter two appear to correspond best to the parameters significantly influencing *pPOX2* induction, namely the amount of oleic acid available per unit of biomass, modalities of oleic acid feeding, and residual oleic acid concentration, since it has been shown that high oleic acid levels can be toxic to the cells [18]. A similar relation between inducer concentration and induction levels has been reported for *pLIP2* induction in the presence of preferred inducers, namely oleic acid and methyl oleate [8,74]. However, more elaborated investigation on feeding strategies remains to be conducted to highlight the optimal conditions of *pLIP2* induction.

For both *pPOX2* and *pLIP2*, hydrophobic inducer dispersion in the culture medium is operated as an emulsion, a technical constraint hampering inducible bioprocesses, particularly at a large scale. A strategy to solve this issue would be to replace part of the hydrophobic substrate by an alternative energy source without affecting recombinant protein yields. In this regard, Sassi and colleagues obtained higher *pLIP2* induction levels by combining oleic acid with glucose as a supplementary carbon source in a shake-flask [76].

Finally, the induction of *pEYK1*-derived promoters has so far only been tested through pulse additions of erythritol and erythrulose during chemostat cultures [31]. Implementation of other induction strategies in fed-batch shall unveil the potentials of these promoters inducible by hydrophilic substrates regarding recombinant protein production. These are under investigation in our laboratory using the CalB lipase from *Candida antarctica* as a model protein.

### 4.2. Culture Medium: a Matter of Definition

Complex and defined culture media seem to be of equal popularity in bioreactor processes. Most of the defined media, though, include a small amount of organic nitrogen, either yeast extract [21,37] or peptone [12,13,14,28], or both [15], to promote high production of recombinant protein [112]. The complex media mainly consist of original or modified versions of yeast peptone dextrose (YPD) and protein production broth (PPB), with the notable exception of a most basic mixture of yeast extract and glucose tested by van Zyl [113]. These media are simple to handle and may lead to higher biomass and protein yields [43], but they suffer from non-defined composition and batch-to-batch variability, explaining why defined media are preferred for controlled applications such as therapeutic protein production [17]. Additionally, since protein-based media could trigger protease gene expression, they are to be handled with care in case the host strain is not deleted for *AEP* and *AXP* genes [120].

Significant yield improvements can be reached through the choice of an adequate culture medium, may it be opting for the most suited predefined medium formula [19], discriminating the best combination of carbon and nitrogen sources [72], or adjusting the nature and concentration of a higher number of nutrients to cell needs via design of experiment-based methods. Prior to bioreactor production, Gasmi and colleagues investigated the effect of minimum medium composition and the nature of nitrogen source and inducer on cell growth and interferon production [17]. The concentration of Pichia trace metal solution (PTM1) was further assessed in a fed-batch reactor, and a subsequent Box–Behnkan statistical experimental design suggested a strong positive influence of vitamins and trace elements, among all PTM1 components, on the activity of enzymes responsible for inducer uptake and/or metabolism. Similarly, Darvishi and colleagues employed Taguchi′s experimental design method to determine the optimal concentration of four major medium components (carbon source, nitrogen source, yeast extract, and thiamine) in a bioreactor [110,111]. Adapting the initial concentration of carbon source may further help to shorten the lag phase inherent to the start of a culture [15].

Along with its adaptability to a broad distribution of biotopes, *Y*. *lipolytica* is able to metabolize a wide variety of carbon sources, of a hydrophobic (e.g., fatty acids and alkanes) or hydrophilic nature (e.g., glucose, fructose, organic acids, and alcohols). It is thus naturally selected as a preferred candidate to valorize alternative carbon sources such as industrial wastes or by-products. For more details, comprehensive reviews on metabolic engineering for alternative substrate utilization have recently been released [121,122]. Taking advantage of low-cost substrates, the production of low added value compounds is susceptible to reaching a break-even point [8]. 

Alternative carbon sources comprise both pure molecules and raw materials or waste, mostly from agricultural or industrial origin. Best yields and productivity are generally obtained with the pure form, whose composition is mastered, in contrast to a complex substrate that contains impurities [30,110,111]. However, in substrate valorization, upper performance levels are not always targeted, provided that the alternative carbon source contributes to rendering the process cost-effective.

The best-known example of alternative carbon source valorization is raw glycerol, a by-product of the biodiesel industry, cheaper than glucose, that has been exploited for recombinant protein production for a long time [12,13,14,15,31,43,112,114,123]. For *pLIP2*-driven expression in a bioreactor, olive oil [72] and industrial residue methyl oleate [8,10] have been used as both carbon source and inducer. Other potential hydrophobic substrates for recombinant protein production include olive mill wastewater [124], waste cooking oil [125], and industrial derivative of tallow [90]. All of these have been assessed for bioconversion, bioaccumulation, or metabolite production.

Utilization of the targeted carbon source may require the overexpression of genes encoding enzymes that perform missing or deficient steps of substrate catabolism. Po1g, a strain optimized for heterologous protein production [19], secretes the invertase Suc2p from *S. cerevisiae*. It has been proved efficient to mobilize sucrose from beet molasses for heterologous laccase production at the bioreactor scale [111]. Previous studies on *SUC2* expression highlighted a retention phenomenon of the synthesized enzymes, as part of invertase activity remains associated with the host cells. Lazar and colleagues exploited simultaneously two different signal peptides for invertase gene expression in batch experiments. Measuring intracellular and extracellular activities, they noted that a substantial invertase fraction remained trapped inside the cells and that the phenomenon was amplified with the use of *XPR2* pre-sequence over the native signal peptide [29]. The same trend had been also observed by Förster and colleagues with native signal peptide used for invertase secretion in fed-batch bioreactor, as most of invertase activity was detected at the cell surface and not in the culture supernatant [28,29]. Even if not initially intended this way, cell-bound activity seems more profitable to the cells than secreted enzymes that could get lost in the culture medium. *SUC2* expression in Po1g and in the study of Förster and colleagues is controlled by the inducible promoter *pXPR2*. According to the comparative study of Lazar and colleagues, comparable biomass and enzyme yields can be obtained under control of the constitutive promoter *pTEF*. More recently, Guo and colleagues employed *pTEF* for constitutive intracellular expression of six cellulolytic enzymes. Although resulting yields were lower than in a glucose-based medium, pretreated cellulose sustained the production of a reasonable amount of recombinant lipase in shake-flasks [126]. Keeping these observations in mind, exploiting engineered strains that have been validated for bioconversion, bioaccumulation, or metabolite production would contribute to reducing the costs of bioreactor operation. Among the possibilities to explore, galactose [127], lactoserum, raw starch [128], inulin [30], and xylose [129] appear to be promising options. 

Antifoam addition to the culture broth will depend on culture medium composition and the subsequent recombinant protein application. Medium-composing proteins (in the so-called complex media) tend to stabilize the foam, while fatty acids rather have an antifoaming action [10]. At first sight, antifoam use may appear indispensable to avoid reactor overflowing and recombinant protein denaturation at the gas–liquid interface [123]. On the other hand, the incorporation of antifoam impedes mass transfer and downstream processing [130], and may have a positive or negative influence on cell physiology [131]. The user will have to balance advantages and disadvantages regarding the envisaged process.

### 4.3. Oxygen Supply: Toward Innovation

The level of dissolved oxygen (DO) in the culture broth informs the cell physiology, the growth stage (i.e., exponential or stationary), and the fitness of a cell population. Oxygen requirements expand during the exponential growth phase, mirroring substrate metabolism by an expanding number of cells, while the reverse decrease at the end of a culture follows reduced metabolic activity. Abnormally high DO levels may reflect cell population starvation. Since *Y. lipolytica* is an obligate aerobe, ensuring sufficient oxygen intake is needed to minimize hyphal morphology (promoted in stress conditions such as low DO levels [82,87,132]) and to achieve correct process rates and yields (as shown by [10,133] for β-galactosidase production). 

Fixed agitation (i.e., impeller rotation speed) and aeration (i.e., air flow) values represent the simplest operating design for processes that are not highly oxygen demanding. They are easy to set up, especially on a large scale, provided that process oxygen requirements are already known and that no unexpected cellular behavior occurs. Otherwise, cells may encounter a severe lack of oxygen [9,111], resulting in the aforementioned consequences. For optimal oxygen transfer to the culture broth (estimated by the oxygen transfer rate, OTR), air flow, and more frequently agitation, are manually or automatedly adjusted. For the latter, system accuracy regarding reaction time and response design are expected, to guarantee a compliant reaction to cell demand. Conversely, in the case of intensively growing cultures, the system could be subject to a very rapid stirring speed [123], which is unrealistic on a large scale and generates hydrodynamic stress to the cells [134]. Oxygen-enriched air [10,12,13,14,15,114] and pure oxygen spikes [18,20] may be implemented to prevent or palliate to agitation and aeration limitations during lab experiments, but these technologies seem barely viable on a large scale. Growth rate may be balanced by the choice of the culture medium composition, to comply with the oxygenation limitations of a system (the more culture medium is concentrated in nutrients, the more metabolic activity, and consequently oxygen demand, will be elevated). One may also think to ponder the use of hydrophobic carbon sources in the culture medium, since hydrophobic substances such as olive oil seem to limit oxygen transfer [135].

Strategies to overcome oxygen limitations at larger scale rely on process engineering—improvement of oxygen solubility in the culture medium—and genetic engineering—improvement of cellular capacity to mobilize oxygen. To enhance *Y. lipolytica* oxygen uptake rates, Bhave and Chattoo expressed a hemoglobin-encoding gene from *Vitreoscilla stercoraria* (Vhb) and monitored the effects on cell growth in oxygen limiting and non-limiting conditions. They selected the operating conditions of 0.05 vvm and 500 rpm, causing a DO drop around 0% after 5–6 h of culture, as oxygen limiting, while the combination of 0.3 vvm and 1000 rpm, resulting in a DO value permanently above 20%, was considered non-limiting [7]. *VHB*-expressing cells performed distinctly better than control cells under oxygen limitation. However, co-production of Vhb and another recombinant protein still needs to be assessed. In most reported studies, the lower accepted DO value is systematically fixed at no less than 20% [18,21,22,23,36,37,43,53,71,110,112,113,123]. Oxygen availability exceeding cell requirements is less damageable since *Y. lipolytica* has proficient coping mechanisms [136,137], paving the way for an oxygen solubility increase in pressurized bioreactors [137,138]. Comparative studies shall determine whether this technology is beneficial to recombinant protein production and is cost-effective on a large scale. Oxygen solubility has further been demonstrated to increase in a biphasic reactor containing ionic liquids such as the efficient oxygen carrier perfluorodecalin in addition to the aqueous culture medium [135]. This approach resulted in a significantly improved oxygen transfer capacity of the system from gas to liquid phase: the volumetric mass transfer coefficient *k_L_a* increased up to 230% from YPD medium without perfluorodecalin to YPD medium containing perfluorodecalin. Such type of biphasic reactor is conceivable on a large scale, especially in strains engineered to tolerate high ionic liquid concentrations [139]. However, the utilization of these ionic liquids may raise environmental concerns given that perfluorodecalin belongs to the perfluorocarbons, a category of compounds decried by the Kyoto Protocol [140].

### 4.4. Multifaceted Implications of pH

Unlike many yeast species, *Y. lipolytica* is able to grow decently under a broad range of pH conditions [141,142]. However, comparably to DO levels, environmental pH values reflect the culture stage and affect recombinant protein production in several ways. Firstly, the synthesis of *Y. lipolytica* extracellular proteases is pH-dependent, AXP being secreted at pH 2–6 and AEP at pH 6–9 [143]. If the recipient strain is not deleted for extracellular proteases, the pH target value must be set around 6 and controlled tightly, to avoid any damages to secreted recombinant proteins, as observed for human interferon [18]. pH also contributes to the induction of some promoters. It is now established that full induction of *pXPR2*, the promoter of the gene coding for AEP, requires a pH of 6.8, suboptimal pH values leading to suboptimal recombinant protein yields [28]. In fed-batch cultures based on *pXPR2*, growth phase can be carried out at a different pH, as long as the pH is adjusted at 6.8 for the production phase [15]. p*LIP2* seems to present the same kind of pH-dependent induction pattern in defined medium, with higher induction levels obtained at higher pH [73]. On the contrary, inducible promoter *pPOX2* does not seem to suffer from pH dependency, and is used more freely between pH 5 [16,17,18] and 6.8 [20] in bioreactors. This characteristic is still to be assessed for the *pEYK1* and *pEYD1* series of promoters, limited for the moment to pH 6.8 [31,77]. pH is also known to be an effector of dimorphic transition. Most studies point out a tendency to adopt a yeast-like morphotype at acidic pH, and a hyphal morphotype at pH close to neutrality. Under defined culture conditions, however, cells may adopt another morphological behavior. Indeed, Timoumi and colleagues observed a predominance of filamentous cells at pH 4.5 and 7 versus ovoid cells at pH 5.6 during batch cultures [91]. Aside from morphological consequences, opting for a low pH value will allow *Y. lipolytica* cells to grow while limiting the risk of bacterial contamination, which is of great industrial interest. Finally, all these considerations are to ponder with pH influence on the recombinant protein, since inadequate pH value may be unfavorable to recombinant protein activity or even detrimental to its stability [43,123].

### 4.5. Striking a Balance with Biomass Load

Surprisingly, little attention has been paid to the initial bioreactor cell load, despite the fact that this parameter directly influences the kinetics of cell growth. Few examples mention an exact value [16,17,84,110,111], and most case studies simply repeat the same preculture conditions for all experimental replicates without more precision. The same minor attention is given to biomass concentration at the start of the protein production phase in the case of processes based on an inducible promoter. Most processes end up the growth phase when the concentration reaches 20–30 g DCW·L^−1^ [12,13,17,19,114], which is considered sufficient to support recombinant protein production, as above-mentioned. To compensate the deleterious consequences of human protein synthesis on cell fitness, Gasmi and colleagues forced the cell concentration to reach 90 g DCW·L^−1^ via exponential glucose feed prior to induction. Despite that, hGM-CSF (human granulocyte-macrophage colony-stimulating factor) production impacted greatly the cell population, dragging it down to 20 g DCW·L^−1^ by the end of the culture [16]. In a unique study, the effect of biomass concentration at the beginning of induction phase was assessed for human interferon production. Higher recombinant protein and biomass yields were obtained when growth phase was ended at 73 g DCW·L^−1^ in comparison with 105 g DCW·L^−1^ [18]. In other biological systems, inconsistent behaviors have been reported following induction [144,145]. For *Y. lipolytica,* as for other microorganisms, more data are required to get a general view of the influence of initial cell load. 

Together with initial cell load, specific growth rate also affects the process outcomes. According to reported studies, protein production may be positively [22] or negatively [21,84,113] affected by high growth rate. In the first case, higher biomass concentration produced higher enzyme overall yields, even if the specific protein production was decreased. In the second case, lower growth rates resulted in increased volumetric and specific protein production. Madzak and colleagues explain this correlation by possible impaired folding and subsequent degradation of complex proteins at high growth rates [84]. An optimal growth rate of 0.1 h^-1^ was determined in chemostat at the beginning of a series of studies on rice α-amylase [13] and used consequently afterwards [12,14]. Ever since, several studies have adopted a value of 0.1 h^-1^, higher than the lowest reported growth rates [21,113] but lower than the average maximal growth rate [16,113,123], however without further investigation [16,18,114].

Arising from initial cell load and growth rate, high cell density cultures are a preferred strategy to optimize recombinant protein volumetric productivity, which is a key parameter of bioprocess cost effectiveness [2,15,112]. High-cell density is favored by sufficient nutrient intake and is thereby typically associated with fed-batch setup [11,12,13,14,15,21,22,23,36,37,112,113]. Yet, in specific cases, highest cell density does not result in best protein yields [19], so a rapid assessment of the most adequate option may significantly contribute to process optimization.

### 4.6. Working Volume: To Navigate along the Scale

Most of the reported studies were carried out in working volumes ranging from 600 mL to 10 L, values that are both large enough to limit the number of experiments and small enough to hinder the exploitability of results for scale-up. To palliate such issues, alternative bioreactor setups exist at both ends of the spectrum. On one hand, parallelized mini-bioreactors offer to screen rapidly a broad number of experimental parameters in small volumes with controlled conditions. They can be operated in batch, fed-batch, or continuous mode and possess miniaturized sensors for culture follow-up. The DASbox^®^ mini bioreactor system for microbiology from Eppendorf (Hamburg, Germany) is suited for a 60–250 mL working volume and was exploited for *Y. lipolytica* cultures aiming to accumulate lipids [93], produce erythrulose [146], or study the regulation of promoter *pEYK1* through *YFP* gene expression [31]. For this last study, the DASbox^®^ system was run on a chemostat mode. Once cell population has reached a steady state, the effect of an external perturbation on the system can be assessed specifically over time. The bioREACTOR system from 2Mag (München, Germany) is suited for an 8–15 mL working volume and has been used in our laboratory for culture medium optimization. On the other hand, switching to industrial volumes implies modifications in the physical behavior of the culture broth, resulting in heterogeneous micro-environments inside the bioreactor that can impede the bioprocess if not correctly managed [134]. So far, the only reported recombinant expression involving *Y. lipolytica* at pilot scale concerns the β-galactosidase, used as a reporter gene in a 500 L reactor [10]. 

### 4.7. Analytical and Predictive Bioprocess Models

Scarcely adopted, bioprocess modeling offers insightful information to decrypt and optimize recombinant cultures. In the course of epoxide hydrolase (EH) fed-batch production, Maharajh and colleagues investigated the effect of several constant glucose feed rates via an analytical model [22]. Under the selected experimental conditions, a second-order polynomial best described DCW evolution over time, and the relationship between specific EH activity and specific glucose feed rate, while a linear relationship was observed between specific glucose feed rate and volumetric EH activity and productivity, and biomass. In a second study, the effect of exponential glucose feed rates was investigated regarding EH production [23]. Changing from a constant to an exponential feed rate modified the relationships between different parameters. For both biomass productivity and yield, a direct relationship was established with exponential glucose feed rate. An inverse relationship was detected between specific EH activity and exponential feed rate and between specific EH activity and biomass yield. The relationships between exponential feed rate and volumetric EH activity and between exponential feed rate and volumetric EH productivity corresponded to second-order polynomials. These responses were introduced in DESIGN-EXPERT 6 software (Stat-Ease, Inc., Minneapolis, MN, USA) to develop an integrated surface response model predicting the optimal exponential glucose feed leading to maximal EH production. Experimental validation of the model confirmed its accuracy (standard deviation was less than 10% between modeled and actual data, except for biomass productivity). Such a methodology could easily be exploited to optimize other fed-batch processes. In another study, Celińska and colleagues sought to unveil the distribution of carbon and nitrogen fluxes during insect α-amylase (SoAMY) fed-batch production [112]. As expected, most of the carbon and nitrogen sources were dedicated to biomass formation and SoAMY production, though part of the resources were diverted to metabolite production (namely erythritol, mannitol, citric acid, and potentially other metabolites not analyzed during the study). Only 45% of the carbon and 36% of the nitrogen sources were recovered through this simplified analysis. The remaining carbon load was most probably released as CO_2_ or incorporated into non-analyzed metabolites, while a high nitrogen load was still present in the culture medium at the end of the process. This kind of calculation shall help to optimize carbon and nitrogen loads in culture media for recombinant protein production. 

### 4.8. Coupling Recombinant Protein Production with Another Bioprocess

Usually, high optimization effort is directed to the production of a sole recombinant protein. To obtain the best protein yields and make the production profitable, it seems preferable to avoid metabolite secretion or their accumulation inside the cells. However, the challenge could be seen in another way, by taking advantage of *Y. lipolytica* multipotency to increase the process rentability, rather than minimizing the formation of by-products. Co-production could include metabolite synthesis, or bioaccumulation in the form of single-cell protein or single-cell oil. This lead has been suggested by Celińska and colleagues [112] for SoAMY production. As seen above, SoAMY secretion was accompanied by sizable titers of erythritol, mannitol, and citric acid that could, according to the authors, “significantly contribute to the process economy”. This co-production principle is seldom explored but could be applied to recombinant enzymes secreted for alternative substrate valorization. For example, sucrose metabolization for citric acid production [28,29] could be coupled with invertase recovery, and inulin metabolization for the formation of single-cell protein [30] could be coupled with inulinase recovery.

## 5. Examples

Thanks to its physiological properties, and as detailed in the above sections, *Y. lipolytica* is a versatile host system for the production of recombinant proteins. Based on these properties, process strategy can easily be adjusted to various applications, from low value industrial enzymes to high value therapeutic proteins. The following examples illustrate these two extreme cases. 

### 5.1. Low Value Protein: The Lipase Lip2p for Industrial Catalysis

The lipase Lip2p is an extracellular glycoprotein encoded by the *LIP2* gene. In the *Y. lipolytica* wild-type strain, the production of Lip2p is triggered in the presence of hydrophobic substrates such as triglycerides, oleic acid, or methyl oleate, the latter being a by-product of the chemical industry [147]. Lipases have various fields of applications [75]. Some of them, such as treatment of fat-containing wastes or incorporation in laundry powder, require low-cost production processes.

To improve Lip2p yields, Fickers and colleagues overexpressed the *LIP2* gene under the control of its own promoter in a recipient strain devoid of glucose catabolite repression [72]. Chromosomal integration of the *pLIP2*-*LIP2* expression cassette was performed both in mono- and multi-copy. The production process was conducted in batch and fed-batch modes at a 20 L scale in a complex, industrial-type, low-cost medium composed of yeast extract, NH_4_Cl, and a glucose or olive oil as a carbon source, with or without addition of tryptone, as the latter was previously reported to enhance *LIP2* expression [74].

In batch cultures, the mono-copy strain JMY 1105 behaved better than the multi-copy strain JMY 1103 in terms of enzyme yields, specific enzyme productivity, and the time of maximal enzyme activity, although biomass yields were lower. For both strains, the medium containing olive oil led to higher specific enzyme productivity compared to the glucose-based medium (1172 versus 252 U·mL^−1^·mg DCW^−1^ for JMY 1105 and 657 versus 45 U·mL^−1^·mg DCW^−1^ for JMY 1103, respectively). Moreover, biomass and enzyme yields were positively influenced by the addition of tryptone to the culture medium, as previously reported [74].

Fed-batch process for lipase production was then developed at a 20 L scale. After 46 h of batch phase, biomass and lipase activity reached 9 g DCW·L^−1^ and 20,000 U·mL^−1^, respectively. The feeding process consisted of a single addition of a mixture of olive oil (20 g/L) and tryptone (10 g·L^−1^). After 80 h of process, biomass and lipase activity reached values of 17 g DCW·L^−1^ and 158,000 U·mL^−1^, respectively. 

Through this fed-batch process, the mono-copy JMY 1105 strain allowed a 6-fold increase in lipase yields compared to batch cultures, a 138-fold increase compared to the parental strain (i.e., LgX64.81), and an increase of several thousand fold regarding the wild-type strain [148]. In order to further improve process yields and reduce production costs, different hydrophobic substrates considered as by-products of the chemical industry were tested for lipase production. The best of these substrates was methyl oleate [149]. As a downstream process, Fickers and colleagues later proposed a simple, cost-effective procedure to formulate a powder of Lip2p [150]. The treatment consisted in four steps, namely continuous centrifugation, filtration on a 0.2 µm plate filter system, ultrafiltration, and dehydration on a spray-drier after the addition of milk powder and gum arabic as powder stabilizers.

### 5.2. High Value Protein: The Human Interferon α2b for Medical Use

The human interferon alpha 2b (hIFN α2b) is a complex cytokine of 19 kDa, comprising two disulfide bonds. It finds application as an antiviral and antineoplastic drug [151]. Similarly to other therapeutic proteins, hIFN α2b could advantageously be produced in *Y. lipolytica* due to the high efficiency of its secretion pathway and the low level of hyperglycosylation of this host yeast [152]. In two successive studies, Gasmi and colleagues investigated heterologous expression optimization in *Y. lipolytica*, using hIFN α2b as a model protein [17,18].

hIFN α2b production was developed in a 5 L bioreactor with strain JMY1852 bearing a single copy of the expression cassette composed of the following parts: *POX2* promoter–*LIP2* signal peptide–codon-optimized hIFN α2b–*LIP2* terminator. A defined culture medium was specifically designed to maximize hIFN α2b yields and to minimize endogenous protease activity. Glucose was used as the main carbon source for cell growth, and oleic acid as an inducer. Growth phase consisted of a 20 h batch culture on a glucose-based medium, followed by an exponential glucose feed after depletion to maintain a specific growth rate of 0.1 h^−1^ for about 20 h more. Surprisingly, higher final biomass and hIFN α2b yields at the end of the production phase were achieved when growth phase was arrested at 73 g DCW·L^−1^ over 105 g DCW·L^−1^. This illustrates that the highest cell density at the beginning of the production phase does not systematically result in the best process performances.

Since growth inhibition was observed during the hIFN α2b production phase (i.e., after the switch from glucose to oleic acid), a constant glucose feeding strategy was implemented in addition to the one-step oleic acid addition. This efficiently promoted both cell growth and hIFN α2b yields. In a second step, the influence of four oleic acid feeding strategies (one-step injections at two concentrations, pulses at regular intervals, and a constant feed at a low rate) was assessed. A constant oleic acid feed rate yielded the highest hIFN α2b concentration in the shortest culture time, despite the lowest biomass concentration. This feeding strategy delivered sufficiently inducing molecules to the cells, while preventing oleic acid accumulation in the culture medium (which can be toxic to the cells).

Prior to yield calculation and biological activity measurement, culture supernatant was concentrated by 8 μm filtration, and the resulting solution was successively chromatographied on a desalting column, a cation exchange column, and a gel permeation column, to obtain purified hIFN α2b.

Through medium and cultivation process optimization, Gasmi and colleagues achieved a 3500-fold increase in hIFN α2b yields and a 25-fold increase in hIFN α2b biological activity regarding initial shake-flasks cultures. These results highlight the adequacy of *Y. lipolytica*-inducible systems for precise recombinant protein production. 

## 6. Conclusions and Prospects

As highlighted in the present review, *Y. lipolytica* can be considered as an efficient cell factory for the production of recombinant proteins. This non-conventional yeast delivers yields comparable to well-established systems such as *S. cerevisiae* and *P. pastoris*. By contrast to *S. cerevisiae*, *Y. lipolytica* processes are not hampered by high hyperglycosylation issues or metabolite diversion toward ethanol production in aerobic conditions. Moreover, *Y. lipolytica* expression systems do not require the utilization of harmful and flammable inducers such as methanol in *P. pastoris*.

Recombinant processes using *Y. lipolytica* have strong advantages. Numerous molecular tools are available to optimize recombinant gene expression, including a variety of promoters, high cell density is easily achievable when coupled with fed-batch mode, and other culture conditions (e.g., complex or defined medium, classical or alternative carbon source, pH value) can be adjusted to considered recombinant protein production thanks to the versatility of *Y. lipolytica*.

However, industrial implementation of *Y. lipolytica*-based processes still suffers from technical issues such as high oxygen requirements, possible by-product formation, and uncontrolled dimorphism. Concerning oxygen supply, innovative approaches have been proposed to increase dissolved oxygen levels (i.e., ionic liquids, pressurized reactors) and *Y. lipolytica* oxygen uptake rate (i.e., *Vitreoscilla stercoraria* hemoglobin expression). In further developments, *Y. lipolytica* weaknesses regarding morphology and by-product formation shall be used as strengths. Rather than minimizing by-product formation, coupling recombinant protein together with metabolite synthesis or bioaccumulation could make the process economic. Recently, dimorphism has been reported as an asset to develop an original reactor setup based on immobilized-cell technology. Currently restricted to succinic acid production, this technology could advantageously be extended to secreted recombinant protein production.

In the attractive host *Y. lipolytica*, simultaneous optimization of strain and process through already available tools will greatly sustain future developments in recombinant protein production.
